# Round the Hospitals

**Published:** 1916-10-07

**Authors:** 


### ROUND THE HOSPITALS.

THE Secretary of State for War announced on Oct. 3 the reconstitution of the Supply of Nurses Committee, together with an alteration in its terms of reference, formerly so vague and unsatisfactory. Lord Knutsford has withdrawn from the Committee, owing, it is stated, to his inability to attend the meetings of the Committee for some time. Lord Knutsford's preoccupations are so numerous, and the work he does for institutions is so onerous, that this inability should have been foreseen before his name was added to the Committee. The remaining members of the old Committee continue to serve, so that the London Hospital is still a good deal over-represented, in spite of Lord Knutsford's withdrawal. Nine new names are added, eight of them being those of well-known matrons, and the ninth that of the Countess of Airlie. Miss Becher, R.R.C., Matron-in-Chief of the Q.A.I.M.N.S., heads the list of those thus added to the Committee, and the others are Miss Sidney Browne, R.R.C., Matron-in-Chief, T.F.N.S.; Miss Haughton, Matron of Guy's Hospital; Miss Cox-

Davies, R.R.C., Matron of the Royal Free Hospital; Miss Lloyd Still, Matron of St. Thomas's Hospital; Miss McIntosh, Matron of St. Bartholomew's Hospital; Miss Gill, R.R.C., Matron of the Royal Infirmary, Edinburgh; and Miss Barton, R.R.C., President Poor-Law Infirmary Matrons' Association, and Principal Matron, No. 3 T.F. General Hospital, London. It will be noticed that all these ladies, except Miss Gill, represent London training schools. Since London is the centre of administrative effort in connection with Army nursing and of the Joint Committee of the Red Cross and the Order of St. John, and since the Committee will meet in London (starting on October 9, we are informed) it is inevitable that London matrons should constitute the bulk of the Committee. It is understood that two at least of the ladies now added declined to be co-opted when approached by the old Committee for that purpose. The terms of reference are now officially given as follows: "The Committee have been appointed for the purpose of ascertaining the resources of the country in trained nurses and women partially trained in nursing, so as to enable it to suggest the most economical method of utilising their services for civil and military purposes."

---

The appointment of Miss Annie Burgess as matron of Crumpsall Infirmary, to succeed Miss Girdlestone, whose coming retirement we announced in our issue of September 9, may come as a surprise to the nursing world, where Miss Burgess is little known at present. She has been 10½ years at Crumpsall, having been trained there, and occupied successively the positions of nurse, ward sister, and assistant matron, and is much liked by the staff. She is considered an excellent teacher, a thoroughly capable woman, and it is confidently hoped that she will maintain the high standard at Crumpsall and its reputation as a great Poor-Law nurse-training school.

---

In our issue of July 15 we announced the coming retirement of Miss H. F. Hopkins, for thirty years the matron of the South Devon and East Cornwall Hospital. This retirement will shortly become effective, and to mark their high appreciation of Miss Hopkins' great kindness to them and as a testimony of their love and gratitude for her unremitting labour in furthering the general welfare of all who have come under her care and guidance the past and present nursing staffs last week presented her with a clock in an inlaid Chippendale case, a pendant, and a cheque for fourteen guineas for the purchase of a writing-desk of her own choice. The presentation was made by Sister Jacob, the senior member of the staff, who is in her twenty-third year's work at the institution. She referred to the many developments which have been seen at the Plymouth institution during the matron's reign, both in connection with the nursing staff and generally. Amongst the most recent are the foundation of the Linen League and a new mortuary chapel, which was opened so recently as the beginning of last month (The Hospital, September 9, p. 537). To obtain both these most necessary complements to the work of a great modern hospital Miss Hopkins has spared no pains, and it must be a supreme satisfaction to her before her departure to have seen the Linen League enter upon its second year of useful existence. Miss Hopkins has had the further satisfaction of seeing seven of her sisters and eight nurses doing military nursing duty, either with Queen Alexandra's or the Territorial Nursing Service, one of whom was in the King's Birthday Honours list, whilst over one hundred Red Cross members have been trained at the South Devon and East Cornwall Hospital. The matron has always taken an active part in keeping the hospital in touch with the latest developments in her department, and has set the staff a high standard of thoroughness in work and devotion to duty.

---

The value to hospitals of Linen Guilds was recently emphasised by Lady Plymouth when speaking at Cardiff to the Needlework and Linen Guilds of King Edward VII.'s Hospital. The supply of garments, useful though it is, is not in itself the only asset. An asset of even greater value is the widespread and living interest which these Guilds create in all that is going on in the hospital, and the opportunity which they give to all who wish to come into direct touch with the institution. In these tragic days it is pleasing to see how the instinct of suppressing self is almost universal, and anything that will foster and develop it is in every sense a benefit to the community. We hope all matrons will encourage and work for the establishment of an active Linen Guild at their institution, believing as we do that once the giver of personal service is won the interest displayed by each in the chosen institution is continuous and increasing.

---

It is frequently urged that it is an anomaly that the teaching of nurses should be carried out by persons who, however competent they may be as nurses, sisters, or superintendents, have not necessarily any qualifications as lecturers or teachers. It is held by some educationists that a university lecturer, who should also be a trained nurse, should be on the staff of each of our great training-schools. This suggestion raises problems that perhaps the establishment of the College of Nursing may help to solve, but it is interesting to note that St. Thomas' Hospital for some time has had a sister-tutor or teaching sister, and the West Ham Union Infirmary has now appointed a sister, whose sole work is to teach. This sister attends the lectures given by the medical staff and the matron, and then holds classes on the various subjects. The classes are kept as small as possible, so that each probationer nurse may get individual attention. Cooking is included in the subjects taught by the teaching sister at West Ham. It would be interesting to know the names of other hospitals or infirmaries having a sister-tutor on the staff, and to compare the examination results at such institutions with others where the teaching and lectures are given by the matron or sisters in the ordinary way.

For Mental Nurses in Military Hospitals, see p. 15. For Matrons' and Sisters' Appointments, see p. 18.

